# The Influence of Selected Properties of Sintered Iron Doped with Lubricants on Its Tribological Properties

**DOI:** 10.3390/ma18174211

**Published:** 2025-09-08

**Authors:** Wiesław Urbaniak, Tomasz Majewski, Grzegorz Śmigielski, Anna Trynda, Aneta D. Petelska

**Affiliations:** 1 Faculty of Mechatronics, Kazimierz Wielki University, Chodkiewicz 30, 85-867 Bydgoszcz, Poland; wurban@ukw.edu.pl (W.U.); gsmigielski@ukw.edu.pl (G.Ś.); 2 Faculty of Mechatronics, Armament and Aerospace, Military University of Technology, Kaliskiego 2, 01-489 Warsaw, Poland; tomasz.majewski@wat.edu.pl; 3 Faculty of Chemistry, University of Bialystok, Ciolkowskiego 1K, 15-245 Bialystok, Poland; a.trynda@uwb.edu.pl

**Keywords:** hexagonal boron nitride, molybdenum disulfide, tungsten disulfide, tribological properties, density and porosity, hardness

## Abstract

This study investigated materials intended for use in porous bearings, incorporating selected layered materials. Previous research has demonstrated that layered compounds, such as molybdenum disulfide (MoS_2_), tungsten disulfide (WS_2_), and hexagonal boron nitride (h-BN), can significantly enhance tribological performance. However, these improvements in tribological properties may be accompanied by undesirable characteristics that could limit the practical application of such materials. Therefore, further investigation was necessary to gain a better understanding of their behavior. To this end, composite materials containing iron (Fe) and varying amounts (0.5, 2.5, and 5 wt%) of layered materials were fabricated using powder metallurgy and sintering techniques. The study evaluated the impact of compaction pressure applied before sintering on the tribological properties and hardness of the materials. Additionally, the long-term stability of the composites was assessed after six years of storage under ambient conditions. The results confirmed that incorporating layered materials into the structure of porous bearing materials improves operating conditions and reduces the coefficient of friction by more than 20%. However, after six years of ambient storage, only the samples containing h-BN remained unchanged. Samples containing WS_2_ or MoS_2_ exhibited partial degradation, with evident signs of corrosion and grain fragmentation.

## 1. Introduction

The development of modern civilization aims to reduce the physical effort required by humans in relation to essential material goods. Automation and robotics are being increasingly implemented. In some cases, entire production lines are fully automated and operated exclusively by robots. The role of machines and mechanical systems in our daily lives is growing exponentially [1]. Most machines and devices are based on the interaction of kinematic pairs, which connect to form tribological nodes. These nodes are points where friction between parts causes overheating, surface wear, and potential damage to the machine. Choosing the right lubricants to reduce friction is essential to minimize machine wear. The importance of lubricants in the economy continues to grow steadily and greatly affects related costs [2]. The limited availability and environmental concerns caused by the use of lubricants require new solutions. An approach is to modify existing lubricants to achieve a very low coefficient of friction and better heat dissipation from the tribological node [3]. This can be carried out, for example, by adding layered materials [4]. Such additives include, among others: molybdenum disulfide (MoS_2_), tungsten disulfide (WS_2_), hexagonal boron nitride (h-BN) [5,6,7,8] or graphite (C), etc. [9,10,11,12,13,14,15,16]. These additives can be incorporated into the lubricating oil or the material of parts that are directly in contact with each other [17,18,19,20]. Although the first method is relatively straightforward, applying layered material to the contact surfaces may provide a more effective way to leverage their lubricating properties [21]. Layered materials are characterized by atoms of individual elements bonded together in layers by strong covalent bonds. Weak van der Waals forces connect the layers [22], allowing them to easily slide over each other and ensuring a very low coefficient of friction [23]. Numerous studies have confirmed the advantages of this mechanism [24,25,26,27]. At the same time, their low production cost, lack of negative environmental impact, and broad range of applications might be even more important. In many ways, the best additive from this group could be h-BN [28], as it offers a relatively low friction coefficient, only slightly higher than MoS_2_, but is cheaper and safer for health. It can be used at higher temperatures than other additives [29,30]. Currently, there are several methods for delivering layered lubricants to a tribological pair. They can be applied during the manufacturing process of interconnected machine components using sintered powder metallurgy [31,32]. They can also be applied directly to the friction surfaces of kinematic pairs by laser coating or even simple painting. A relatively simple method for delivering lubricity-enhancing materials is the use of lubricating mixtures enriched with such materials [33], which can also be self-lubricating metal matrix composites [34].

Extreme operating conditions—such as high temperatures, elevated pressures, and aggressive chemical environments—often make the use of liquid lubricants difficult. In such cases, solid lubricants can offer an effective solution. They are used in applications such as space technology, where extreme environmental conditions are present [35], as well as in electric vehicles.

The primary purpose of layered lubricant additives is to reduce the coefficient of friction, but in some cases, their operational properties may deteriorate. This deterioration may be caused by water adsorption on the surfaces of their grains [36], which at elevated temperatures can promote the formation of sulfur compounds. For example, used layer lubricants with h-BN do not cause this problem.

The patterns observed in the study [30] suggest that the tribological properties of these materials may be influenced by their hardness. In addition, changes appeared on the surfaces of these materials just a few days after preparation, with clear signs of corrosion observed. Therefore, this study aimed to investigate how factors such as hardness, porosity, and chemical composition (specifically, the content of lubricant additives) affect the tribological properties and storage capacity of self-lubricating sintered bearings. These bearings were made using powder metallurgy from iron powder (Fe), along with additives such as MoS_2_, WS_2_, and h-BN, under normal warehouse conditions.

## 2. Materials and Methods

### 2.1. Testing Materials

The test samples were sinters made from iron powder with varying amounts of lubricants added to the mixture. Basic information about the materials used is presented below.

#### 2.1.1. Iron Powder

The base material for the test samples is S.C. 100.40 iron powder (99.9%), manufactured by Högänas AB, Högänas, Sweden. It is a popular and widely available powder used in the production of components through sintered powder metallurgy. It is a material of high chemical purity, characterized by a spongy structure and excellent compactability (Table 1).

#### 2.1.2. Lubricant Additives

To improve the lubricating properties of the test samples, selected layered materials were incorporated into the material before pressing:

- Hexagonal boron nitride (h-BN, 99.9%) with an average particle size of 0.5 μm and 1.5 μm;
- Molybdenum disulfide (MoS_2_, 99.9%) with an average particle size of 1.5 μm;
- Tungsten disulfide (WS_2_, 99.9%) with an average particle size of 0.6 μm.

Hexagonal boron nitride (h-BN) is an inorganic chemical compound obtained synthetically. It was first obtained by William H. Balmain in 1842 (see more in Table S1 and Figures S1 and S2 in Supplementary Material) [37]. Currently, it is produced by forming boron-nitrogen bonds in high-energy processes. It is the cheapest of the layered materials mentioned here. It exists in three varieties (α, β, γ), each with different crystallographic structures and properties. In tribology, h-BN with a hexagonal structure is primarily used. This variety is characterized by a structure composed of plates of nitrogen and boron atoms bonded together by weak van der Waals forces, strongly covalently bound. This material is widely used, and despite being known for almost 200 years, it has widespread and promising applications. It is chemically very durable and environmentally friendly.

Molybdenum disulfide (MoS_2_) is an inorganic compound found in nature as the mineral molybdenite (see more in Table S2 and Figures S3 and S4 in the Supplementary Material) [37]. It is characterized by a low friction coefficient (as low as 0.19) [38]. At elevated temperatures (350 °C), it can oxidize to molybdenum trioxide (MoO_3_), which is accompanied by the release of sulfur oxides (SO_x_), which contribute to the deterioration of the lubrication properties. It is insoluble in water, but its decomposition in aggressive environments at elevated temperatures can promote the formation of corrosion sites.

Tungsten disulfide (WS_2_) is an inorganic chemical compound found in nature as the mineral tungstite (see more in Table S3 and Figures S5 and S6 in Supplementary Material) [37]. However, it is sporadic and has a different crystal structure from synthetic WS_2_. Most commercially available WS_2_ is produced through synthetic processes, often using sulfur and tungsten. It occurs in two allotropic forms: hexagonal and trigonal. Tungsten disulfide belongs to the group of materials characterized by the lowest dry friction coefficient, 0.07. The layered crystal structure and high thermal stability are its distinguishing characteristics.

### 2.2. Sample Preparation and Test Procedures

#### Preparation of Samples for Testing

**Figure 1:**
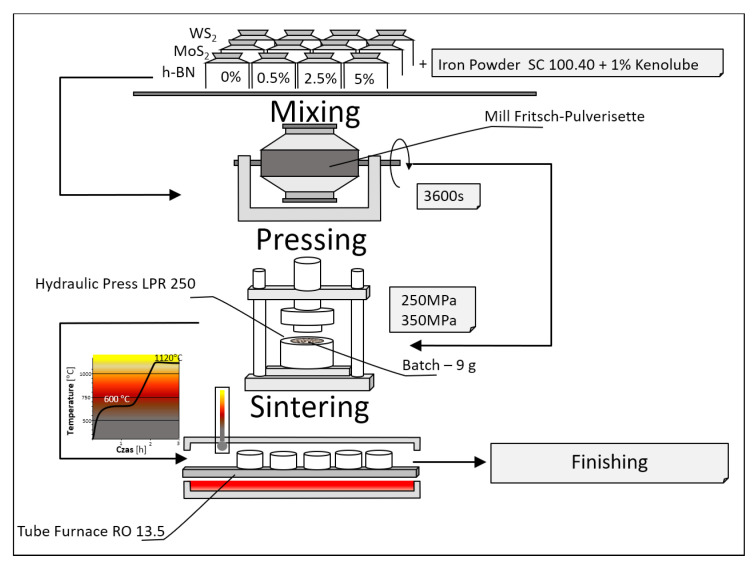
Process of research sample production.

The test samples were prepared using the following procedure (Figure 1): powder samples containing iron with added lubricants were pressed. In this case, hexagonal boron nitride (h-BN) at 0.5 μm or 1.5 μm, molybdenum disulfide (MoS_2_) at 1.5 μm, and tungsten disulfide (WS_2_) at 0.6 μm were used. The powder mixtures included concentrations of lubricant additive of 0%, 0.5%, 2.5%, and 5% by weight. To further reduce the frictional forces between the powder particles and the walls of the die and punches (as well as between particles), the lubricant Kenolube (99.9%, Höganäs Sweden AB) was added at 1% by weight. It was assumed that the test samples would have two porosity levels: approximately 17% and approximately 26%. Therefore, the pressing conditions were pre-defined to achieve samples with the expected porosity, ensuring that material porosity did not influence subsequent test results.

Appropriate amounts of powder mixtures were prepared and subjected to high-energy mixing in a Fritsch-Pulverisette ball mill, type 05-202, manufactured by Fritsch, Idar-Oberstein, Germany (see more in Figure S7 in the Supplementary Material) for one hour. This mixing process was intended to homogenize the chemical composition of the mixtures throughout their entire volume and to pre-combine the powder particles of different components (through intense friction), preventing their segregation during transfer to the next test station. The process was conducted in ethyl alcohol (96%, Avantor Performance Materials Poland S.A., Gliwice, Poland) to avoid the oxidation of the powders. The powders were dried in a JEIO TECH OV4-65 vacuum dryer. A vacuum of −0.06 MPa and a temperature of 100 °C were applied.

The next step was to produce the molded parts using the die pressing method. An LPR 250 hydraulic press (Testchem, Radlin, Poland, see more in Figure S8 in Supplementary Material) was used for this purpose. The molded parts weighed approximately 9 g. The pressing pressure was approximately 250 MPa for samples with an assumed porosity of 26% and approximately 350 MPa for samples with an assumed porosity of 17%. The selection of the pressing pressure was carried out experimentally in such a way as to obtain densities as close as possible to the assumed ones. In the remainder of this article, the designations 26% and 17% were conventionally adopted (to distinguish the samples).

The finished compacts were subjected to density testing and then sintered in a tube furnace (RO 13.5 from VEB Electro-Industrieofenbau Römhild, Römhild, Germany; see more in Figure S9 in Supplementary Material) in a protective atmosphere of dissociated NH_3_ (Avantor Performance Materials Poland S.A., Gliwice, Poland). A temperature stop of 600 °C for 1 h was applied. This was intended to remove any lubricant residue from the sinters and ensure a stable oxide reduction process on the powder particle surfaces before the porosity closed, allowing them to be trapped within the sinters. The final sintering parameters were 1120 °C and 1 h.

### 2.3. Research Procedures

#### 2.3.1. Density and Porosity Tests

The density of the sinter was determined using the hydrostatic method, under the PN-EN ISO 2738:1999 standard(https://www.iso.org/standard/21494.html) (accessed on 1 July 2024). The volume of the material is determined by comparing its mass in air with the mass of the material immersed in a liquid of known density. To avoid wetting, the samples were protected with a thin layer of petroleum jelly before measurement in water.

The porosity was calculated using the measured density and the theoretical density of the sintered materials tested.

#### 2.3.2. Hardness Tests

Hardness testing was performed using the Brinell method, which is recommended for sintered and porous materials. This method uses a steel ball as an indenter. After pressing, two diameter measurements were taken. Based on these measurements, the sample’s hardness was determined. A PRIDE UHT 910 hardness tester (Eagle Eyes Quality Inspection Co., Ltd., Zhangzhou, China; see more in Figure S10 in the Supplementary Material) was used for the test. The following test parameters were adopted: indenter (ball) diameter, 2.5 mm; force application time, 15 s; and load, 15.625 kg.

#### 2.3.3. UNMT Surface Layer Characterization Test Kit

Tribological studies were conducted using the UNMT Surface Layer Characterization Test Kit (Bruke, Fairfax County, VA, USA; see Figure S11 in the Supplementary Material for more information). This kit is used for a comprehensive assessment of the mechanical and tribological properties of thin films and solid materials. The included nanoanalyzer enables the evaluation of surface morphology at the nanoscale (nanoimaging), and the measurement of nanohardness and Young’s modulus in the ranges of 1–100 GPa and 10–1000 GPa, respectively, for thin films as thin as 1 nm. The included drives, force sensors, and fixtures allow for micro- and macro-hardness measurements (Vickers, Rockwell, and instrumental methods) as well as micro- and macro-scratch tests over a wide load range (from 0.1 to 2000 N). The test drive (see Figure S12 in the Supplementary Material) enables the system to perform tribological tests in both the aforementioned rotary motion (0.1–1000 rpm) and reciprocating motion (0.1–25 mm stroke, 0.1–25 Hz frequency). The system is equipped with several additional sensors, including a precise capacitive displacement sensor with a resolution of 50 nm, an acoustic emission sensor, an electrical resistance sensor, and temperature and humidity sensors.

#### 2.3.4. Tribological Measurements

Friction measurements were performed on 26 pairs of samples in a ball-on-plane configuration for reciprocating motion, without lubrication, according to ASTM G-133 (https://store.astm.org/g0133-05r10.html) (accessed on 1 July 2024).

The following experimental parameters were used:

- A 3.175 mm (1/8”) diameter ball made of tungsten carbide (WC);
- Load—2.79 N, to ensure pressure under the ASTM G-133 standard;
- Amplitude—2 mm, resulting from the ball used;
- Test duration—1000 s;
- Frequency—5 Hz;
- Total number of cycles—5000;
- Friction path—20 m, resulting from the reduced amplitude.

The tests allowed us to determine the friction forces between the sample and the counter-sample (WC ball), which in turn were used to determine the coefficient of friction. At the same time, the wear resistance was determined based on the resulting wear scar. For this purpose, the width of the resulting groove was measured along and across the scar. The arithmetic mean of the two measurements was used as the result. To assess wear scars, a Nikon ECLIPSE LV100 Optical Microscope (Nikon, Tokyo, Japan; see more in Figure S13 in the Supplementary Material) was used. The microscope was equipped with an NIS-AR computer image analyzer and a scanning stage that allowed movement in the X, Y, and Z axes. The surface assessment after the friction process was performed using the bright field technique.

## 3. Results and Discussion

The numerous studies conducted yielded very interesting results; however, the sheer volume of studies does not allow their inclusion here. Only those that are crucial for evaluating the research and confirming the assumptions made will be cited. Some of the results were published in [30]; only Supplementary Information will be provided here to further illustrate the current state of research. Twenty-six sintered samples were prepared for the study, and the results indicate that the samples obtained (under the same preparation conditions) differed in their final porosity and density.

### 3.1. Density and Porosity Measurement Results

Table 2 summarizes the samples that underwent further testing. It shows that the type of layered material influenced the density and final porosity of the sample.

**Figure 2:**
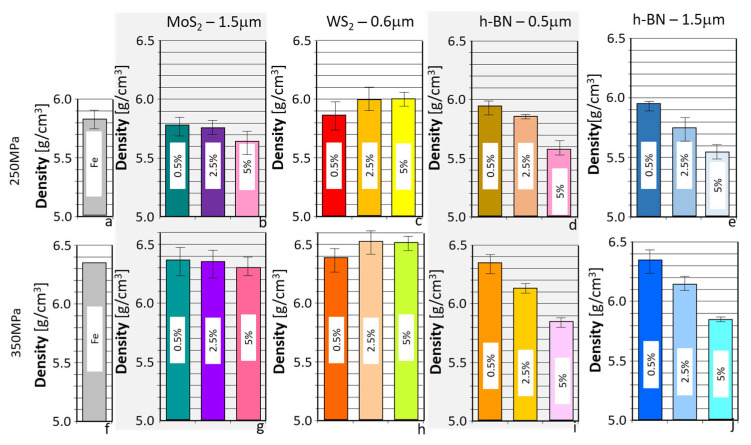
Densities of the samples used for testing. (**a**) Fe; (**b**) Fe + MoS_2_—1.5 µm; (**c**) Fe + WS_2_—0.6 µm; (**d**) Fe + h-BN—0.5 µm; (**e**) Fe + h-BN—1.5 µm at 250 MPa and (**f**) Fe; (**g**) Fe + MoS_2_—1.5 µm; (**h**) Fe + WS_2_—0.6 µm; (**i**) Fe + h-BN—0.5 µm; (**j**) Fe + h-BN—1.5 µm at 350 MPa.

**Figure 3:**
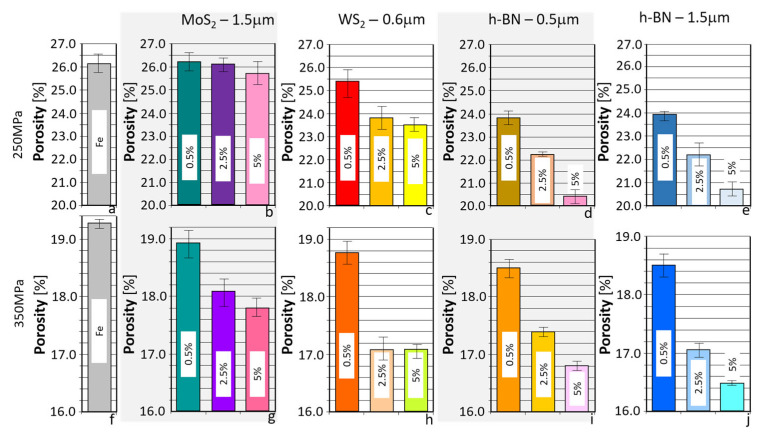
Porosity of samples used for testing. (**a**) Fe; (**b**) Fe + MoS_2_—1.5 µm; (**c**) Fe + WS_2_—0.6 µm; (**d**) Fe + h-BN—0.5 µm; (**e**) Fe + h-BN—1.5 µm at 250 MPa and (**f**) Fe; (**g**) Fe + MoS_2_—1.5 µm; (**h**) Fe + WS_2_—0.6 µm; (**i**) Fe + h-BN—0.5 µm; (**j**) Fe + h-BN—1.5 µm at 350 MPa.

As shown in the table, for a pressing pressure of 250 MPa, samples with porosities ranging from 20.4% to 26.2% and densities ranging from 5.74 g/cm^3^ to 5.99 g/cm^3^ were obtained, for a pressing pressure of 350 MPa, higher density and lower porosity ranging from 16.5 to 19.3% and density ranging from 6.13 to 6.51 g/cm^3^ were obtained. The results presented in the table above (Table 2) are illustrated in the graphs in Figure 2 and Figure 3.

As can be seen from the results quoted, only in the case of WS_2_ was the material obtained with a higher density than in the case of the reference material (without an additive). However, in the remaining instances, lower density values can be observed. Still, with the increase in the amount of additive, the density decreased, most intensively in the case of h-BN.

The presented results indicate that when h-BN was added, similar density and porosity values were obtained for both 0.5 μm and 1.5 μm powders, suggesting that the h-BN particle size did not affect the values of the parameters tested. However, it was found that increasing the amount of additives in the sinter in each case resulted in a decrease in porosity. This suggests that the layered additives act as lubricants in the pressing process, improving the pressing conditions and, consequently, reducing the porosity of the final sinters. It can also be observed that increasing the pressing pressure increases the density and reduces porosity. Generally, the highest porosity was obtained for Fe sinters, and the lowest for Fe + h-BN sinters.

### 3.2. Hardness Test Results

During the hardness measurements of the samples used, the results obtained are presented in Table 3.

**Figure 4:**
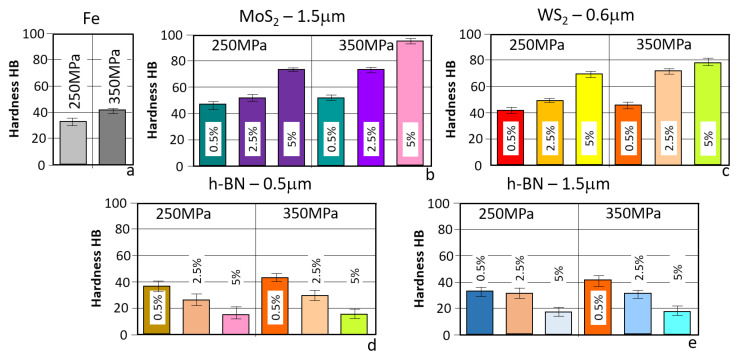
The average results obtained from the hardness measurements. (**a**) Fe; (**b**) Fe + MoS_2_—1.5 µm; (**c**) Fe + WS_2_—0.6 µm; (**d**) Fe + h-BN—0.5 µm; (**e**) Fe + h-BN—1.5 µm at 250 MPa and 350 MPa.

A graphical comparison of the values of the HB hardness measurement results for all the samples prepared for testing is presented in Figure 4.

Analyzing the results presented above, the introduction of layered additives and the sample preparation method have a significant impact on their values. It is noteworthy that in the case of MoS_2_ and WS_2_, increasing their content promotes higher hardness (up to twice as much). Similarly, samples with higher density obtained by pressing at a pressure of 350 MPa exhibit higher hardness.

The situation is somewhat different for samples containing h-BN as a layered additive. As can be seen, the particle size of h-BN has no significant impact on the hardness results obtained. However, the amount of h-BN introduced had a considerable effect on the hardness reduction compared to the reference sample. There is a clear correlation with the density test results (also for MoS_2_ and WS_2_), which allows us to conclude that increasing the density of the materials increases their hardness.

As can be seen, the introduction of a layered additive can have a significant impact on the properties of the resulting materials, enabling the design of materials with the desired properties. The reduction in hardness of samples containing h-BN may come as a surprise, as it can promote the wear of the friction surfaces. The physical properties of h-BN itself may have an impact on the reduction in hardness, i.e., high thermal conductivity, and high melting point (a vast difference between Fe and h-BN), which makes it challenging to form adhesive bonds with iron particles.

### 3.3. Tribological Test Results

*Evaluation of the change in the friction coefficient and wear marks for samples without lubricant additives*.

**Figure 5:**
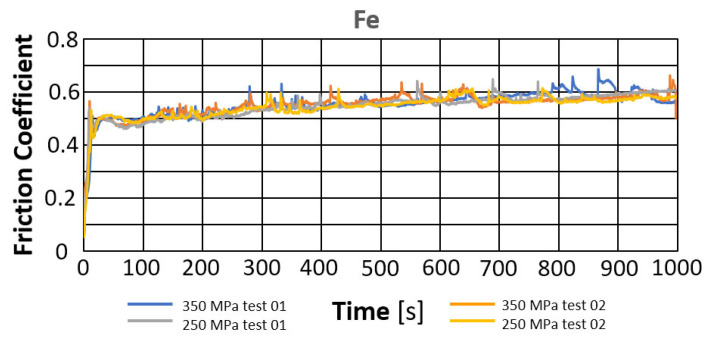
A change in the friction coefficient over time for Fe samples.

The reference point for assessing the change in the coefficient of friction during the tests was the results obtained for a sample made of pure iron (Fe) powder with the addition of the Kenolube lubricant, which was removed during annealing. Figure 5 presents the change in the friction coefficient over time for sintered samples pressed at pressures of 250 MPa and 350 MPa. The average values of the friction coefficient are summarized in Table 4.

Based on the presented curves, the friction coefficients for all runs are similar, reaching values of approximately 0.5 after a brief start-up period. Further testing shows that the friction coefficient value, although with minor deviations, falls within the range of 0.55–0.56, showing a slight upward trend.

**Figure 6:**
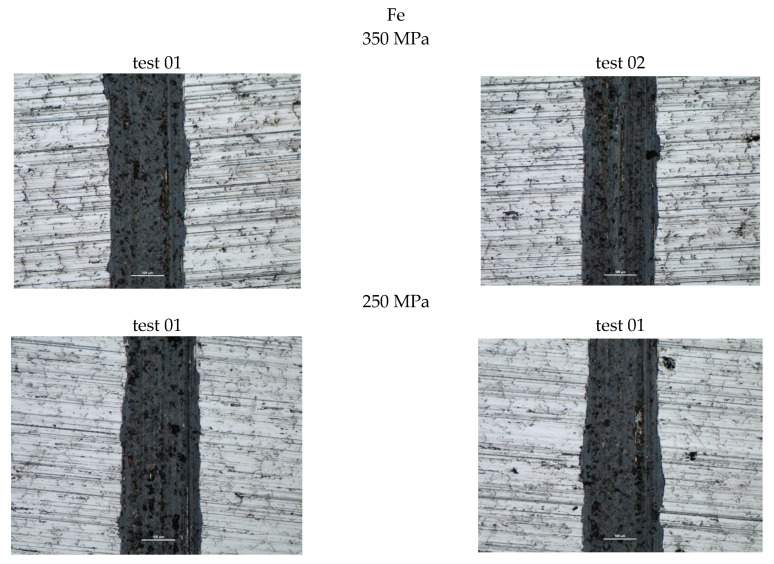
Selected abrasion marks on samples, bright field, scale 100 µm.

Figure 6 also presents photographs of the wear marks obtained on samples containing Fe. Based on the assessment of the marks, it can be concluded that they exhibit typical signs of abrasive wear, as evidenced by scratches parallel to the ball’s direction of motion. Discoloration within the wear marks may indicate oxidation of the sample material. Similar widths characterize all the marks obtained in this manner. The measurement results are summarized in Table 4.

Slightly higher wear was observed in the sample with higher porosity, obtained during the sintering of iron powder pressed at a pressure of 250 MPa (porosity approximately 26%). The comparison showed that a higher coefficient of friction was recorded for the samples pressed at a pressure of 350 MPa.

#### 3.3.1. Samples with MoS_2_ Addition

*Evaluation of the change in friction coefficient and wear marks for sintered steel samples in which 1.5 µm MoS_2_ was used as a lubricant additive in amounts of 0.5%, 2.5%, and 5% by weight.*

**Figure 7:**
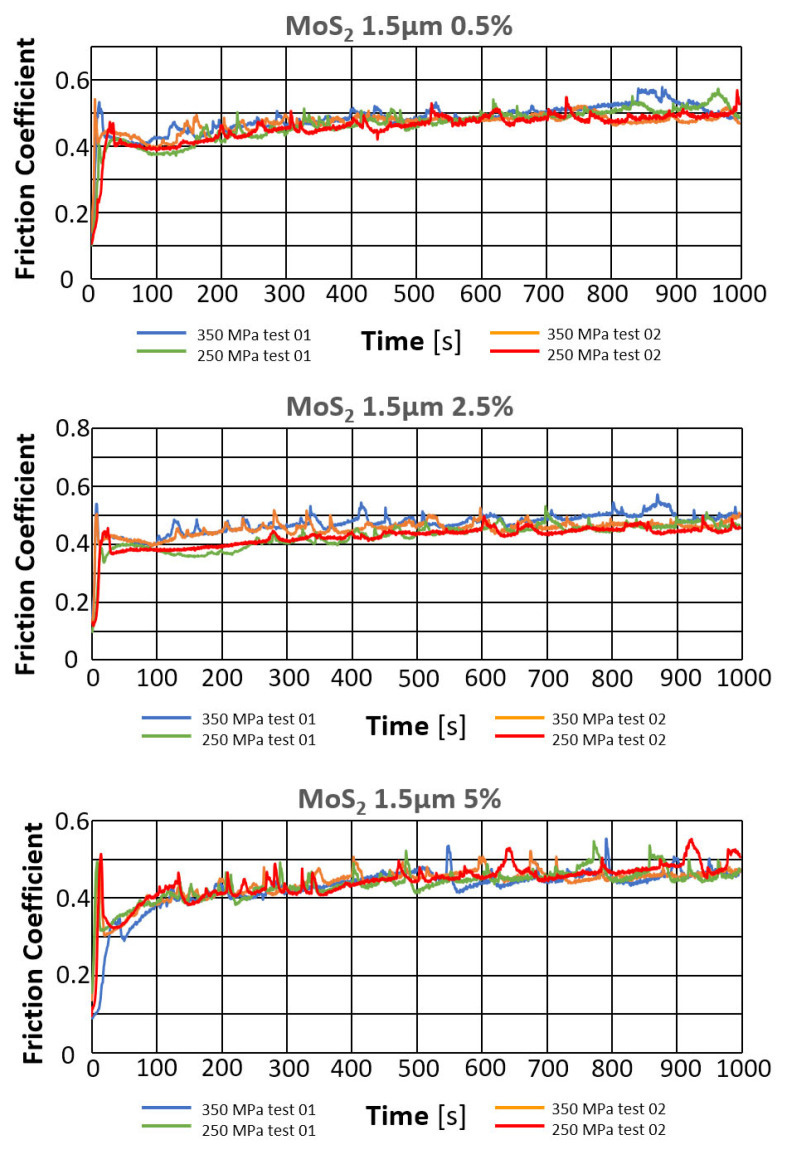
Changes in the friction coefficient over time for samples containing MoS_2_.

The results of the tribological tests, as described in point 2.3.4, for samples containing the MOS_2_ lubricant additive with a grain size of 1.5 µm in amounts of 0.5%, 2.5%, and 5% for both sinter porosities, are presented in the graphs in Figure 7. The obtained results are summarized in Table 5.

**Figure 8:**
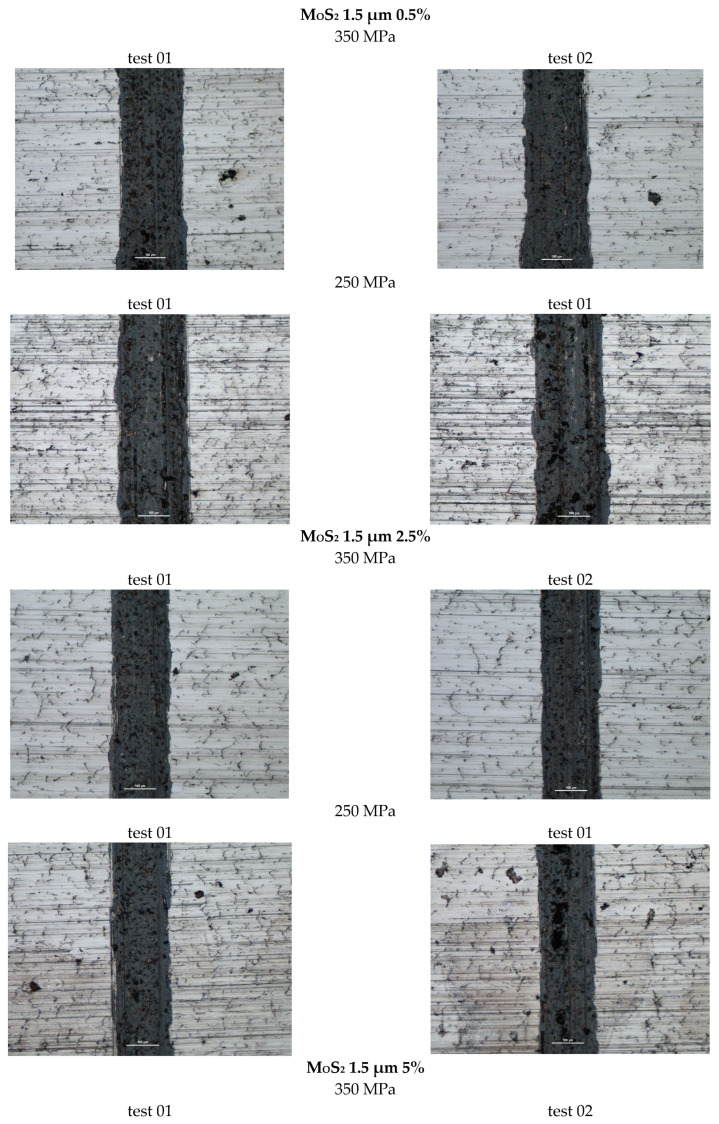

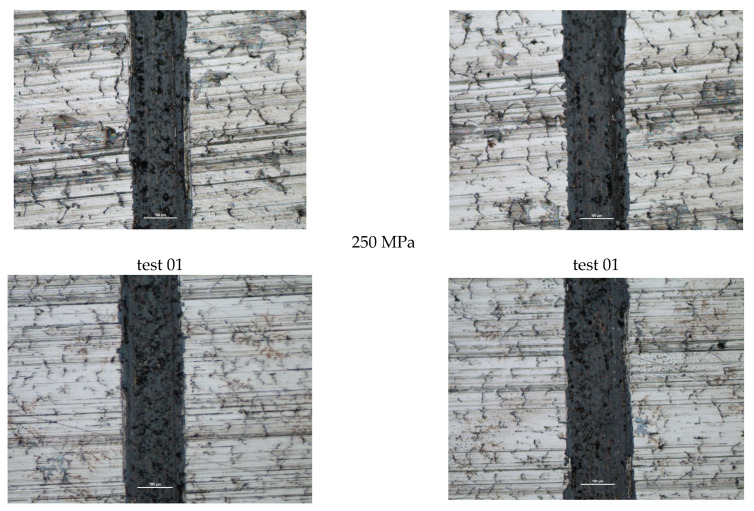
Selected abrasion marks on 1.5 µm MoS_2_ samples (the light line corresponds to the 100 µm scale).

Figure 8 shows photographs of wear marks on samples with a MOS_2_ lubricant additive, having a grain size of 1.5 µm, in amounts of 0.5%, 2.5%, and 5% by weight, for both sinter porosities.

The table below presents the average values of the friction coefficient and wear marks for sintered samples pressed at a pressure of 250 MPa and 350 MPa, containing 0.5%, 2.5%, and 5% MoS_2_, with a particle size of 1.5 μm.

**Figure 9:**
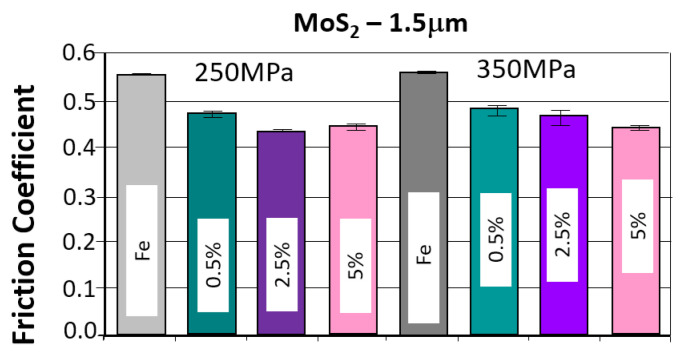
The average friction coefficient values for MoS_2_ samples.

**Figure 10:**
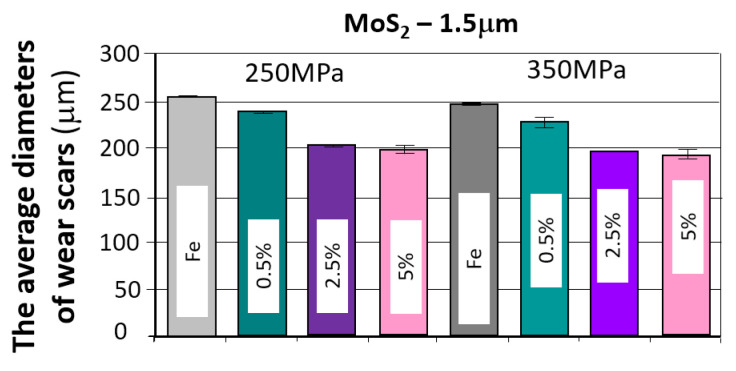
The average values of the diameters of the wear scars for the samples with MoS_2_ addition.

The comparison of the obtained friction coefficient values and wear traces for sintered, pressed samples containing WS_2_ with those made of Fe without the additive is presented in Figure 9 and Figure 10.

Based on the presented curves (Figure 7), a comparison of the samples’ behavior with the MoS_2_ additive can be made. As can be seen, the effect of the lubricant additive on friction compared to the reference material is clear. The friction coefficient versus time curves for all samples with the MoS_2_ additive were similar, regardless of the additive concentration and sinter porosity. Figure 9 shows the effect of the MoS_2_ additive on the average friction coefficient. The lowest friction coefficient was obtained for the sample with 26% porosity (250 MPa) and 2.5% lubricant content (4.30), while the highest was for the sample with 17% porosity (350 MPa) and 0.5% lubricant content. The introduction of the MoS_2_ lubricant additive allows for a material with a 22% lower friction coefficient than the reference material.

The resulting traces, shown in Figure 8, show signs of abrasive wear (scratches parallel to the direction of motion of the ball). Dark gray discoloration within the trace may indicate oxidation of the sample material, and the traces exhibit a relatively constant width.

The addition of molybdenum disulfide to the sinter had a positive effect on wear resistance. With increasing additive concentration, the sinters exhibited increased wear resistance—the effect was particularly pronounced in the 0–2.5% concentration range (a slight increase in wear resistance occurred between 2.5% and 5%. A 5% molybdenum disulfide addition resulted in over 20% less wear compared to the base material (without the additive). An effect of sample porosity on wear resistance was also observed—for both the base material and the sinters with MoS_2_ addition, samples with a porosity of 17% (250 MPa) exhibited slightly higher wear resistance (with fewer wear scars). It was also observed that increasing the MoS_2_ content favored achieving fewer minor wear scars, which, as can be seen, does not coincide with the effect of decreasing motion resistance.

#### 3.3.2. Samples with Added WS_2_

*Evaluation of the change in friction coefficient and wear marks for sintered steel samples in which 0.6 µm WS2 was used as a lubricant additive in amounts of 0.5%, 2.5%, and 5% by weight*.

**Figure 11:**
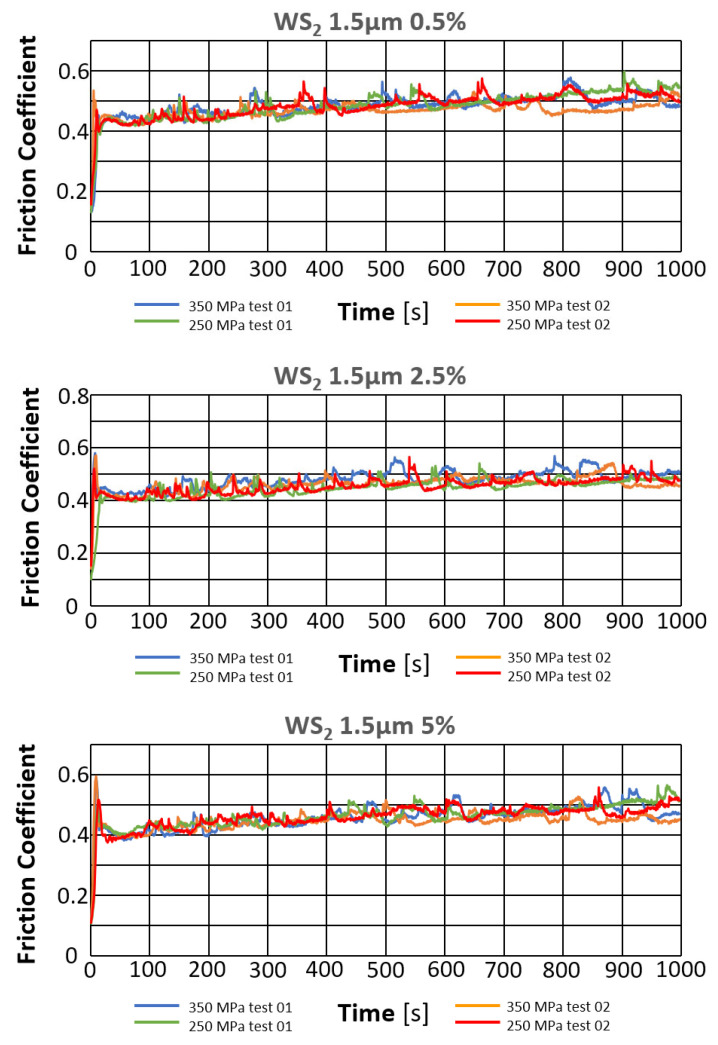
Changes in the friction coefficient over time of samples containing WS_2_.

The results of the tribological tests carried out, as described in point 2.3.4, on the samples containing WS_2_ are presented in the graphs in Figure 11.

**Figure 12:**
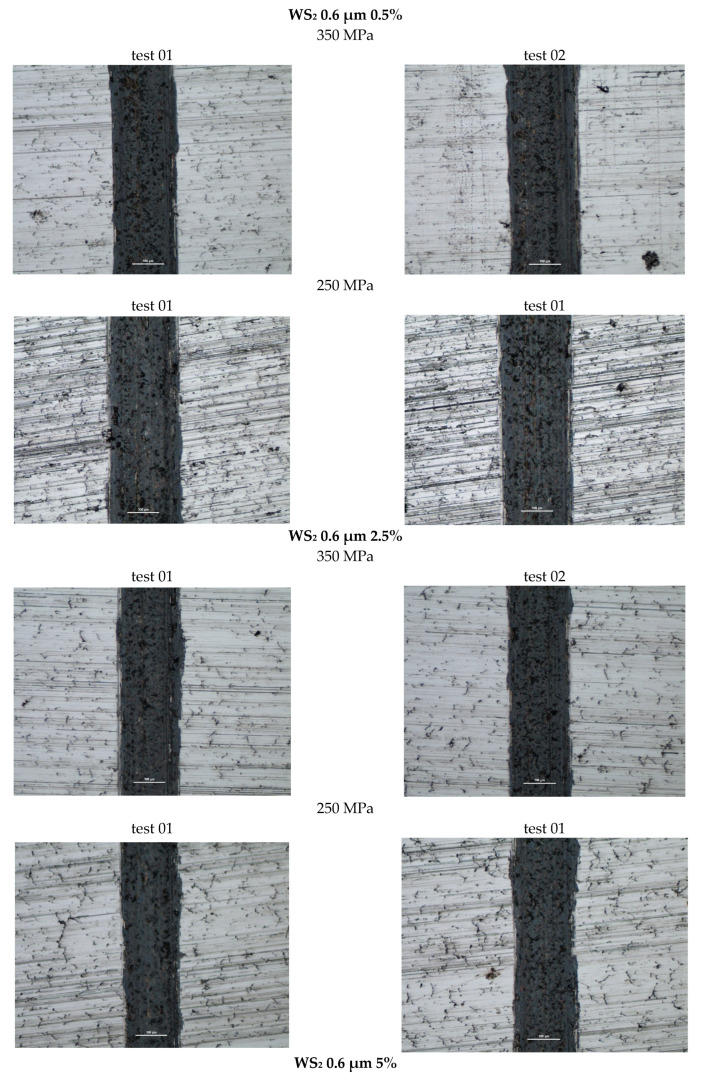

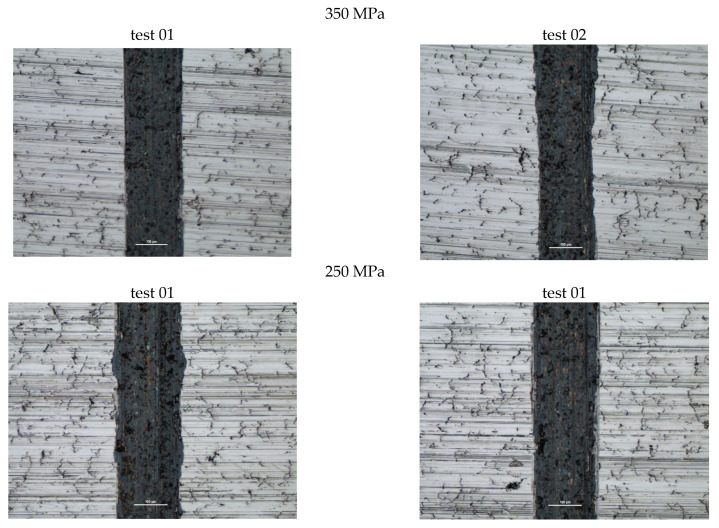
Selected abrasion marks on WS_2_ 0.6 µm samples (the light line corresponds to the 100 µm scale).

Figure 12 presents photographs of the wear marks obtained in samples with the WS_2_ lubricant additive, grain size 0.6 µm, in amounts of 0.5%, 2.5%, and 5% by weight, for both sinter porosities. The marks obtained show signs of abrasive wear (scratches parallel to the ball’s direction of motion). As with pure iron, they show typical signs of abrasive wear, as evidenced by the scratches formed parallel to the ball’s direction of motion. Discoloration appearing within the wear marks may indicate material oxidation. The marks have a relatively constant width.

Table 6 presents the average values of the friction coefficient and wear marks for sintered samples pressed at pressures of 250 MPa and 350 MPa, containing 0.5%, 2.5%, and 5% WS_2_ with a particle size of 0.6 μm.

**Figure 13:**
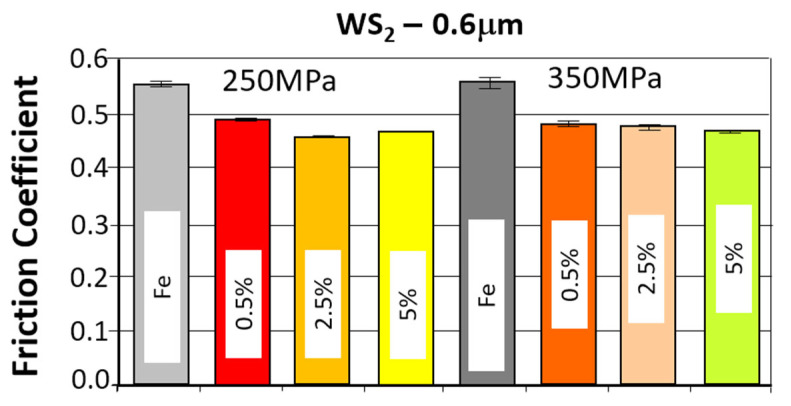
The average friction coefficient values for the WS_2_ samples.

**Figure 14:**
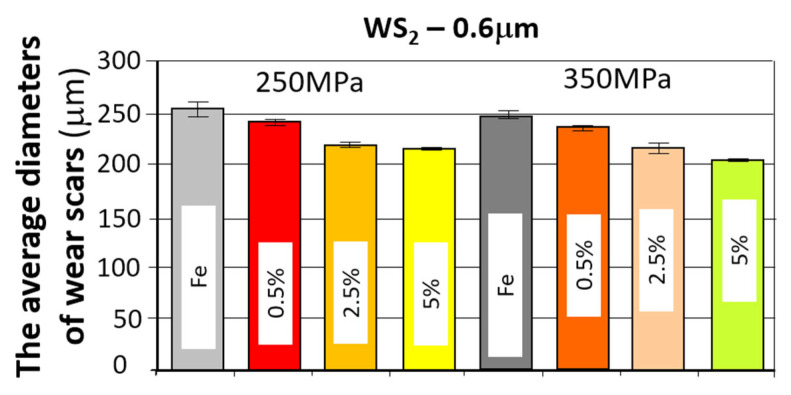
The average values of the diameters of wear scars for the samples with the addition of WS_2_.

The comparison of the obtained friction coefficient values and wear traces for sintered, pressed samples containing WS_2_ with those made of Fe without the additive is presented in Figure 13 and Figure 14.

Based on the curves shown in the graphs, the friction coefficients between the WC ball and the samples made with the WS_2_ 0.6 µm lubricant additive range from 0.4 to 0.6. The time history for the four tests is similar. Shortly after the start of the test, the friction coefficient reached values of approximately 0.55 for the samples with 0.5% by weight of the additive, and 0.6 for the other two types. The change in friction coefficient values was characterized by instability throughout the test, reaching a friction coefficient value of 0.4 for samples with 2.5% and 5% by weight of the additive in the first minute after start-up, and 0.45 for the sample with 0.5% of the additive. The further course of the test indicates that stabilization occurs approximately 500 s into the test duration. The friction coefficient remains in the range of 0.45–0.6 for samples with 0.5% of the additive, in the range of 4.5–5.5 for samples with 2.5% of the additive, and 0.45–0.6 for samples with 5% of the additive.

An apparent positive effect of the lubricant additive on resistance to motion can be observed. For all tested samples with this additive, there is a tendency for resistance to motion to decrease with increasing percentage of the additive (from approximately 0.48 to approximately 0.45). The lowest coefficient of friction was obtained for the sample with ~26% porosity (250 MPa) and 2.5% lubricant additive content (4.53), while the highest was obtained for the sample with ~26% porosity and 0.5% additive content. The introduction of the WS_2_ additive reduces the coefficient of friction by approximately 17%.

Analyzing the wear patterns, appearance, and geometric parameters of all samples with the WS_2_ additive revealed similar characteristics. Abrasive wear predominated, as evidenced by scratches parallel to the ball’s motion. For comparison purposes, the graphs (Figure 9 and Figure 10) also present the results obtained for reference samples (Fe), without the addition of layered lubricants.

Introducing the WS_2_ additive to the sinter had a positive effect on wear resistance. With increasing additive concentration, the sinters exhibited a greater wear resistance. The addition of a 5% tungsten disulfide additive resulted in nearly 20% less wear compared to the starting material Fe. An effect of sample porosity on wear resistance was also observed—for both the starting material and the sinters with the WS_2_ additive, samples with a porosity of 17% (350 MPa) exhibited slightly higher wear resistance (smaller wear patterns).

#### 3.3.3. Samples with h-BN 0.5 μm Added

*Evaluation of the change in friction coefficient and wear marks for sintered steel samples in which h-BN 0.5 µm was used as a lubricant in amounts of 0.5%, 2.5%, and 5% by weight.*

**Figure 15:**
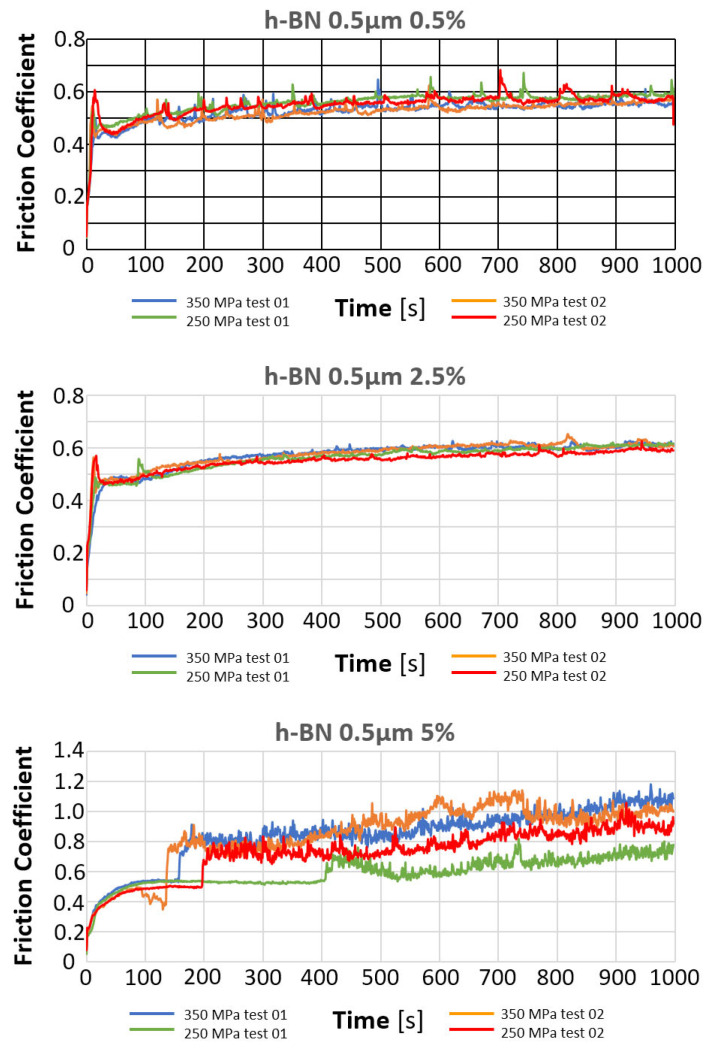
Changes in the friction coefficient over time of samples containing h-BN 0.5 µm.

The results of the tribological tests carried out, as described in point 2.3.4, in samples containing the h-BN lubricant additive with a grain size of 0.5 µm in amounts of 0.5%, 2.5%, and 5% for both sinter porosities, are presented in the graphs in Figure 15.

**Figure 16:**
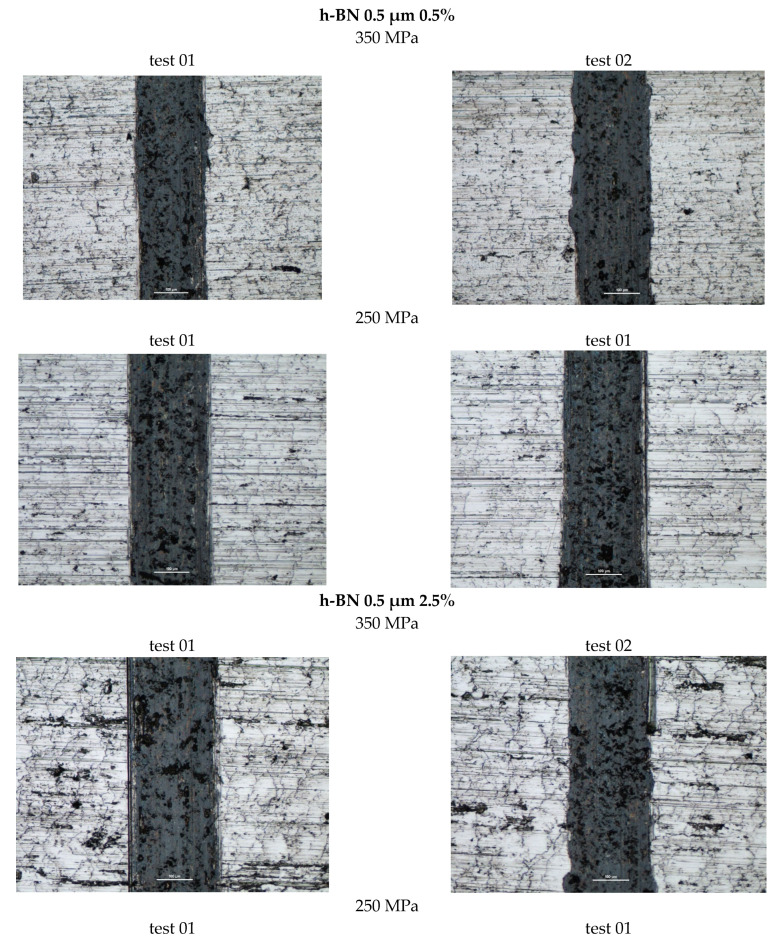

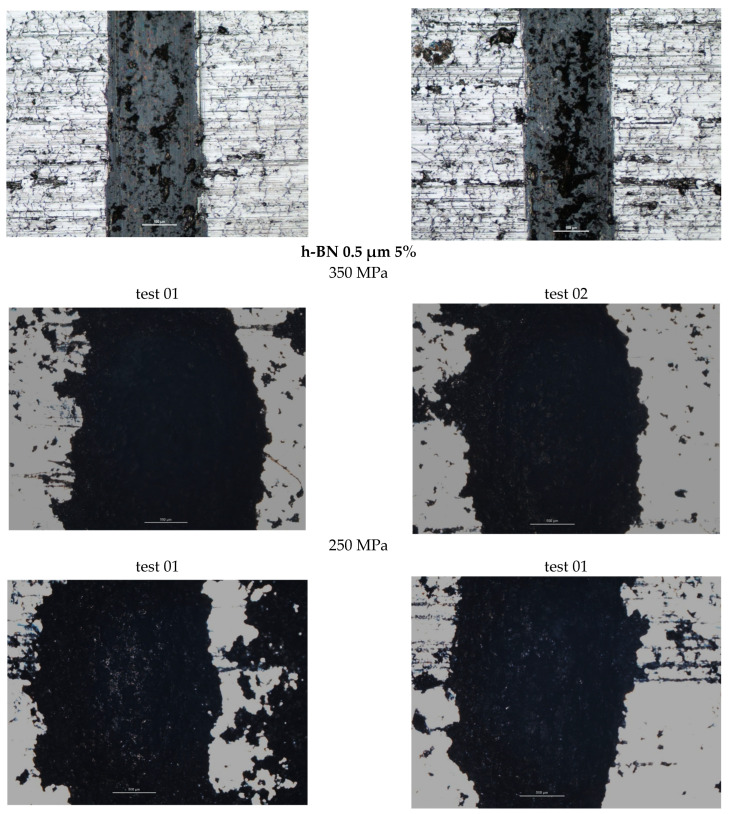
Selected abrasion marks on h-BN 0.5 µm samples (the light line corresponds to the 100 µm scale).

Figure 16 shows photographs of wear traces in samples with an h-BN lubricant additive, having a grain size of 0.5 µm and in amounts of 0.5%, 2.5%, and 5% by weight, for both sinter porosities.

The table below (Table 7) presents the average values of the friction coefficient and wear marks for sintered samples pressed at a pressure of 250 MPa and 350 MPa, containing 0.5%, 2.5%, and 5% h-BN (0.5 μm).

**Figure 17:**
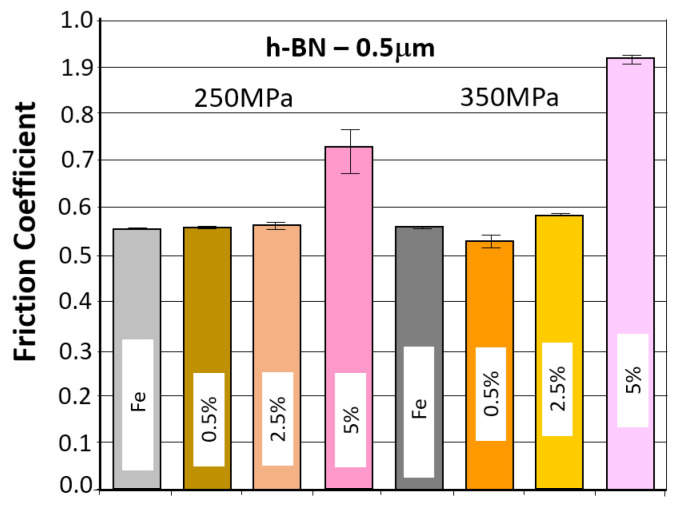
The average friction coefficient values for h-BN 0.5 μm samples.

**Figure 18:**
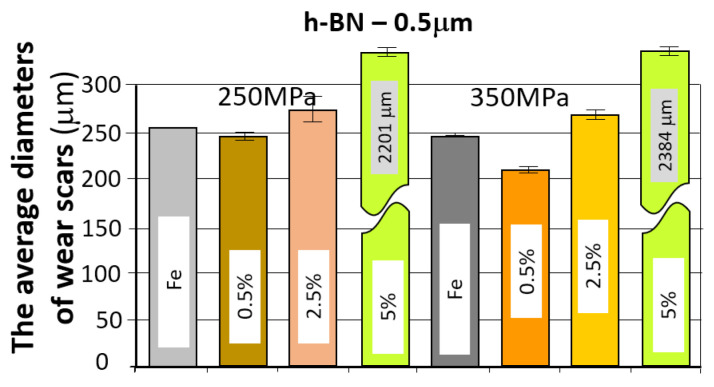
The average values of the diameters of the wear scars for the samples with the addition of h-BN 0.5 μm.

The comparison of the obtained friction coefficient values and wear traces for sintered, pressed samples containing h-BN 0.5 μm with those made of Fe without additive is presented in Figure 17 and Figure 18.

Based on the presented curves, the coefficients of friction between the WC ball and the 0.5 µm 0.5% h-BN samples reach values in the range of 0.4–0.7. The nature of the changes in the friction coefficient over time for both samples (four tests) is similar. Shortly after the test began, the coefficient of friction reached a value of approximately 0.45. Up to approximately 300 s into the test, the coefficient of friction slowly increased to values of 0.5–0.55, after which it stabilized. There were some local fluctuations (peaks) in the coefficient value, although the average value remained relatively constant. For the samples with 0.5 µm 2.5% h-BN, the coefficients of friction reached values in the range of 0.5–0.6. The nature of these changes over time for both samples (four tests) is similar. Shortly after the test began, the coefficient of friction reached values of approximately 0.45 to 0.5. Up to approximately 500 s into the test, the coefficient of friction slowly increased to a value of 0.55–0.6, after which it stabilized. The friction coefficient curves were characterized by noticeably less instability than for samples containing 0.5% h-BN. For samples containing 0.5 µm h-BN with a 5% content, the friction coefficient reached values in the range of 0.5–1.1. The pattern of its changes over time for both samples (four tests) is similar. By approximately 50 s into the test, the friction coefficient values reached 0.5. After approximately 150–200 s of testing (in one case after 400 s), a sudden increase in resistance to motion occurred (friction coefficients in the range of 0.8–1.1). Such high coefficient values and high instability indicate very intense wear (scuffing) processes. The h-BN sample with a porosity of 26% (250 MPa) had a noticeably lower average friction coefficient value.

The resulting wear marks, as seen in Figure 16, show signs of abrasive wear (scratches parallel to the ball’s direction of motion); a dark gray color within the mark may indicate oxidation of the sample material. A relatively constant width characterizes the marks. For material containing 5% h-BN, the obtained marks show signs of very intense abrasive and adhesive wear (material tear). Irregular shapes and variable widths characterize the marks. Averaged wear mark widths are summarized in Table 7 due to the irregularity of the marks, the obtained results should be considered estimates. The sample with a lower porosity of 17% (350 MPa) exhibited slightly higher wear.

When the results obtained from the friction coefficient test depended on the amount and type of lubricant additive, it can be concluded that essentially all of them have a positive effect on achieving a lower friction coefficient compared to the native material. Properly selecting such an additive can reduce the friction coefficient by up to approximately 20%. The most favorable results are achieved with a 2.5% lubricant additive content. A smaller or larger amount has minimal effect on this value.

#### 3.3.4. Samples with h-BN 1.5 μm Added

*Evaluation of the change in friction coefficient and wear marks for sintered steel samples in which h-BN 1.5 µm was used as a lubricant additive in amounts of 0.5%, 2.5%, and 5% by weight.*

**Figure 19:**
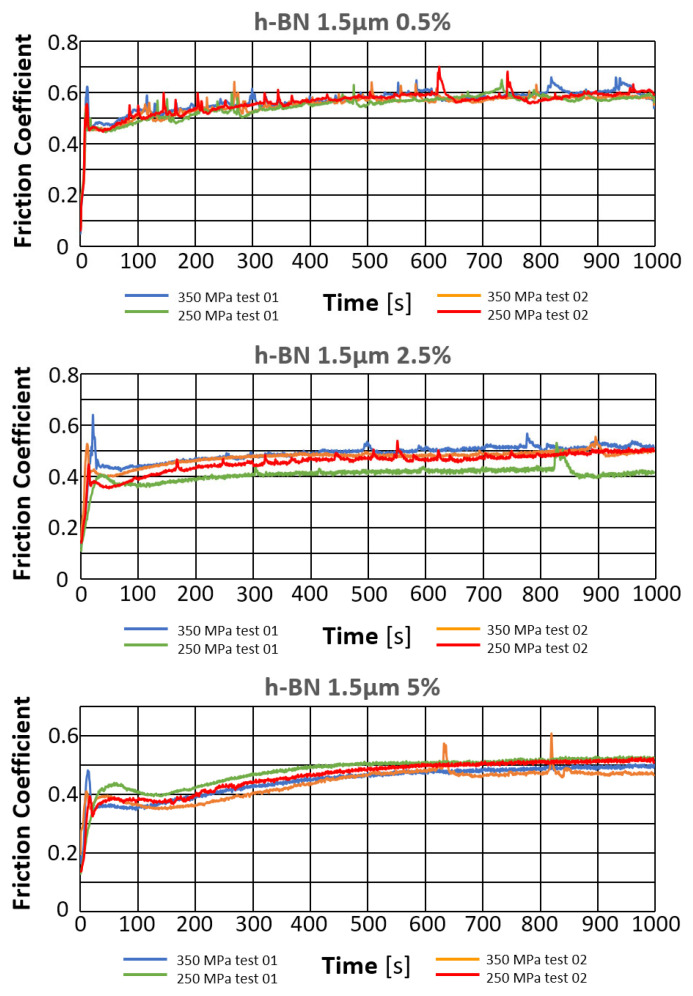
Changes in the friction coefficient over time of samples containing h-BN 1.5 µm.

The results of the tribological tests carried out as described in point 2.3.4 for samples containing the h-BN lubricant additive with a grain size of 1.5 µm, in amounts of 0.5%, 2.5%, and 5%, for both sinter porosities, are presented in the graphs in Figure 19.

**Figure 20:**
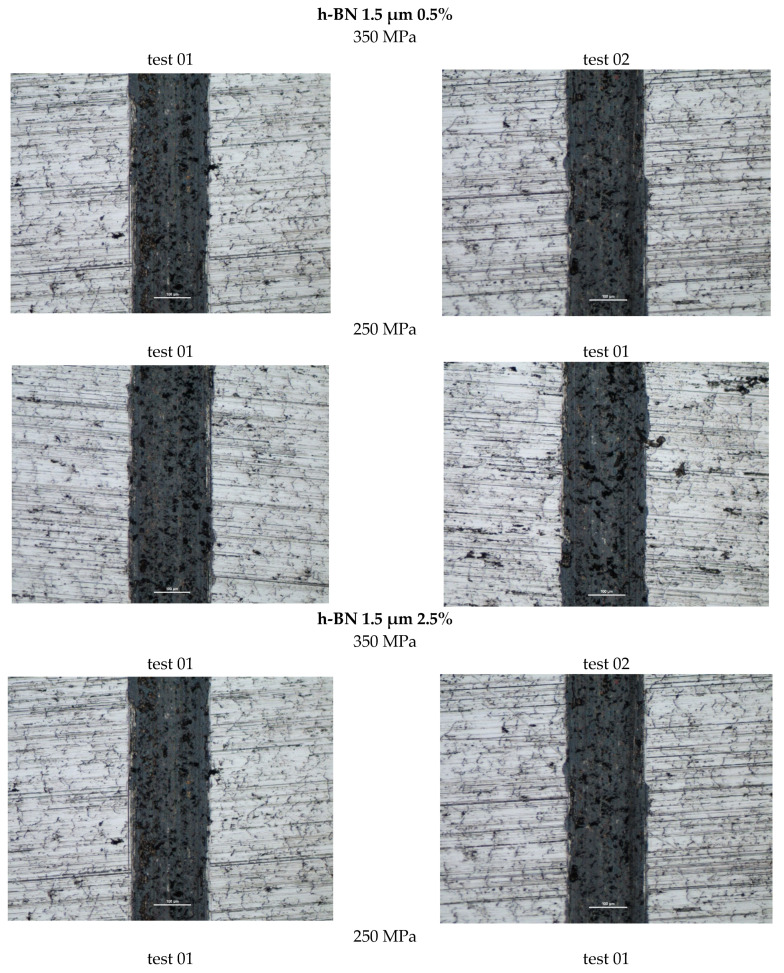

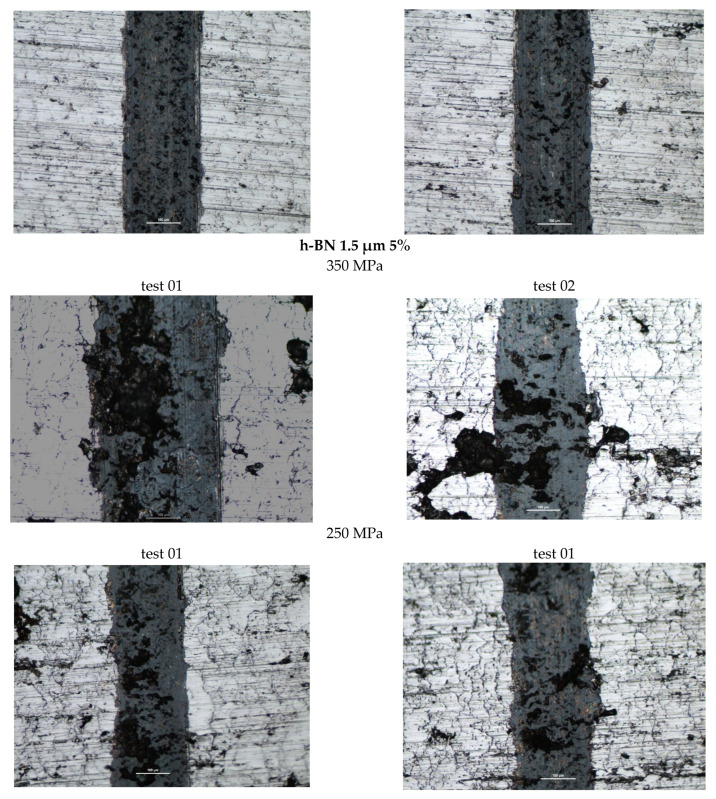
Selected abrasion marks on h-BN 1.5 µm samples (the light line corresponds to the 100 µm scale).

Figure 20 shows photographs of wear marks on samples with an h-BN lubricant additive, having a grain size of 1.5 µm and in amounts of 0.5%, 2.5%, and 5% by weight, for both sinter porosities.

The table below (Table 8) presents the average values of the friction coefficient and wear marks for sintered samples pressed at a pressure of 250 MPa and 350 MPa, containing 0.5%, 2.5%, and 5% h-BN (1.5 μm).

**Figure 21:**
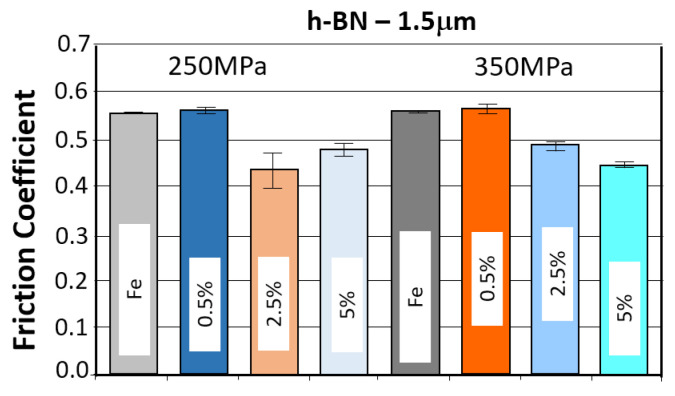
The average friction coefficient values for h-BN 1.5 μm samples.

**Figure 22:**
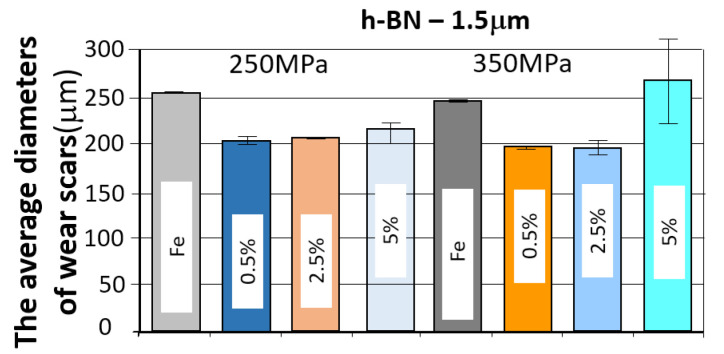
The average values of the diameters of the wear scars for the samples with the addition of h-BN 1.5 μm.

The comparison of the obtained friction coefficient values and wear traces for sintered, pressed samples containing h-BN 1.5 μm with those made of Fe without the additive is presented in Figure 21 and Figure 22.

Based on the presented curves, the friction coefficients between the WC ball and the samples containing 0.5% h-BN 1.5 µm reach values in the range of 0.45–0.7. The nature of the changes in the coefficient of friction over time for both samples (four tests) is similar. Shortly after the test began, the coefficient of friction reached values of approximately 0.45–0.5. Up to approximately 500 s into the test, there was a slow increase in the coefficient of friction to a value of roughly 0.6, after which it stabilized. Some instability characterized the coefficient of friction values (jumps in value). In the case of samples containing 2.5% h-BN 1.5 µm, they reach values in the range of 0.35–0.6. The nature of the changes in the coefficient of friction over time for both samples (four tests) is similar. Shortly after the start of the test, the coefficient of friction reached values of approximately 0.35–0.45. In the latter part of the test, the friction coefficient value increased to approximately 0.4–0.5. The friction coefficient time histories were relatively stable (single jumps in value) with a noticeable upward trend throughout the test. For samples containing 5% h-BN 1.5 µm, they reached values in the range of 0.35–0.5. The nature of changes in the friction coefficient over time for both samples (four tests) is similar. Shortly after the test began, the friction coefficient reached values of approximately 0.35–0.4. In the latter part of the test, the friction coefficient value increased to approximately 0.45–0.5. At the end of the tests, approximately 700 s after the start, the resistance to motion stabilized. The friction coefficient time histories were relatively stable (single jumps in value) with a noticeable upward trend throughout the test. The average values of the friction coefficient for all tests are summarized and presented in Table 8 A noticeably lower average value of the friction coefficient was observed for the WC ball–h-BN sample pair with a porosity of 17% (350 MPa).

The coefficient of friction curves for all samples with h-BN addition were quite similar: the highest resistance to motion was observed in samples with 2.5% h-BN, regardless of grain size, as well as in samples with 5% h-BN with a grain size of 1.5 µm. Particularly undesirable friction behavior was observed in samples with 5% h-BN and a grain size of 0.5 µm, for both sinter porosities. Friction tests were unstable, involving intense wear and even scuffing.

Figure 21 and Figure 22 present the effect of concentration of h-BN additive (for individual powder grain sizes) on the average coefficient of friction. The introduction of the h-BN lubricant additive, combined with the appropriate material selection and manufacturing process, enables a 21% reduction in the coefficient of friction. The resulting wear marks show signs of abrasive wear (scratches parallel to the ball’s direction of motion). A dark gray color within the trace may indicate oxidation of the sample material. The traces are relatively constant in width, with only samples having a porosity of 17% (350 MPa) and a lubricant additive content of 5% for both porosities showing significantly greater signs of wear.

#### 3.3.5. Comparison of the Results of the Tribological Test

When comparing the obtained friction coefficient test results depending on the amount and type of lubricant additive, essentially all of them have a positive impact on achieving a lower friction coefficient compared to the base material. Proper selection of such an additive can result in a reduction in the friction coefficient of up to approximately 20%. The most favorable results are achieved with a 2.5% lubricant additive content. A smaller or larger amount has minimal effect on this value. The situation is similar to the grain size of the lubricant additive powder. An interesting case that requires further analysis is the use of h-BN additive, especially with a grain size of 0.5 μm. The observations suggest that the poorer results obtained in this case may be attributed to issues encountered during the pressing and sintering of iron powder with h-BN additive.

**Figure 23:**
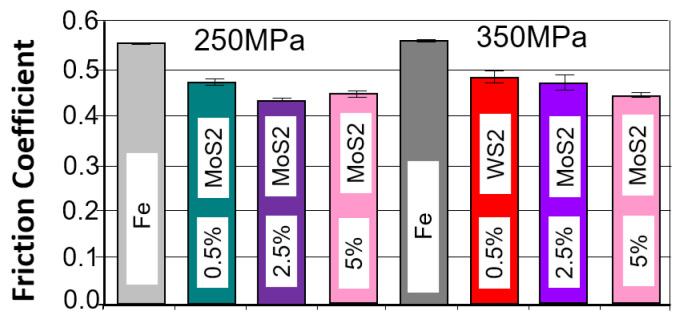
A sample with the lowest friction coefficient in the groups; amount of layer additive in percent.

**Figure 24:**
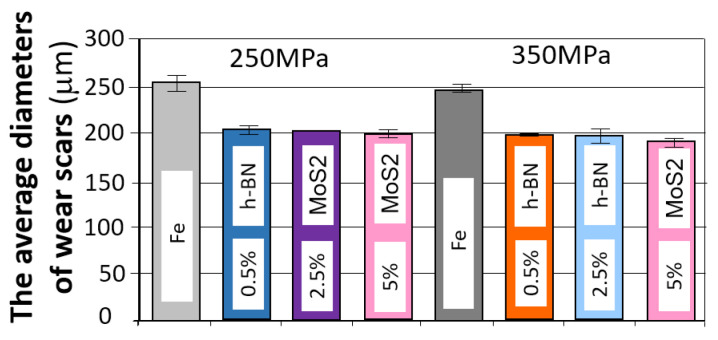
The average values of the diameters of the wear scars in the groups; amount of layer additive in percent.

A summary of the test results for samples that achieved the lowest friction coefficient and the fewest abrasion marks, grouped by type and weight percentage of the layer additive, is presented in Figure 23 and Figure 24.

As can be seen in the graphs, the introduction of a layered lubricant additive helps reduce the coefficient of friction; a properly selected composition can guarantee a reduction in the coefficient of friction by more than 20%. Although the differences are slight, the samples containing MoS_2_ demonstrate the best results in this regard.

When comparing the obtained wear scar results, the best results were obtained for MoS_2_, and in some cases, for h-BN, although the results for h-BN are somewhat ambiguous. This may be due to the strong correlation with strength properties, including hardness. Increasing the amount of h-BN promotes a decrease in density, which in this case is accompanied by a reduction in porosity, unlike the other two additives. At the same time, hardness decreases, resulting in an increased wear scar.

### 3.4. Assessment of the Appearance of Samples After Storage

**Figure 25:**
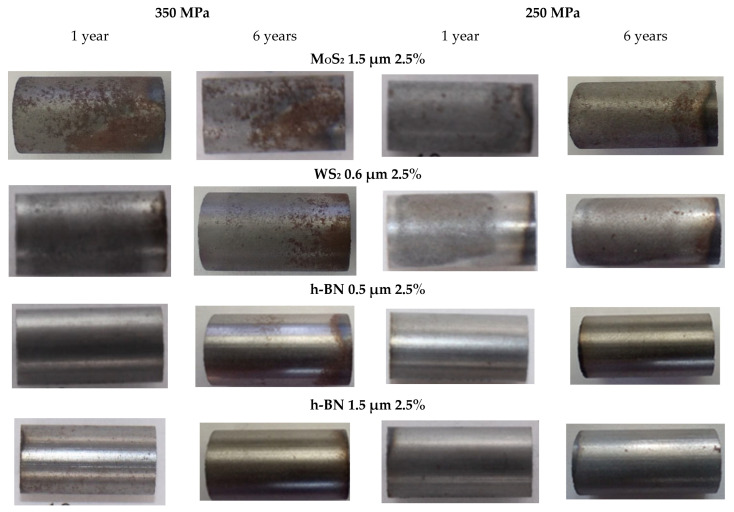
Photos of selected samples after 1 year and 6 years of storage.

The photos below (Figure 25) show some of the samples to show their appearance after one and six years of storage in the laboratory under normal atmospheric conditions (20–23 °C and 40–60% humidity).

As observed, and confirmed by the photos attached in Figure 25 significant oxidation of the sample material occurred on the surface of the samples containing the MoS_2_ and WS_2_ additives. Samples containing h-BN appear significantly better in this regard. Metal powder can be seen in the containers containing samples containing MoS_2_ and WS_2_, which may indicate significant degradation of the sample material. This may be because in the presence of oxygen and water vapor contained in atmospheric air, the oxidation process of MoS_2_ takes place quite quickly [39], and similarly, WS_2_:

2MoS_2_ + 4H_2_O + 9O_2_ → 2MoO_3_+ 4H_2_SO_4_

2WS_2_ + 4H_2_O + 9O_2_ → 2WO_3_ + 4H_2_SO_4_

As a result, the lamellar structure is destroyed and the lubricating properties are lost. Moreover, the sulfuric acid that results from the reaction has a destructive effect on the entire material. At the same time, a higher proportion of layered material promotes increased corrosion. No such changes were observed in the samples containing h-BN; their appearance was similar to that of samples made without the lubricant. On the basis of these observations, samples made of iron powder containing h-BN are significantly better suited for storage. In contrast, samples with MoS_2_ and WS_2_ additives require corrosion protection.

## 4. Conclusions

The studies carried out in conjunction with those previously presented [30] aimed to provide a broader evaluation of how specific additives in layered materials, their amounts, and particle sizes influence specific properties, mainly hardness, tribological characteristics, and storage stability.

Sintered compacts with two different compaction levels (250 MPa and 350 MPa) were used as the test material. The samples were made from pure iron powder and Fe iron powder (SC100.24), sometimes containing layered materials such as MoS_2_, WS_2_, and h-BN.

The introduction of layered materials positively affected tribological properties, decreasing friction by up to 20% in certain instances. The higher hardness of materials containing sulfides guaranteed a slightly lower coefficient of friction.

Analyzing the results of studies that supplement those previously published [30], which aim to more accurately evaluate the effect of hardness, porosity, and chemical composition of the materials on their tribological properties and storage susceptibility, the following conclusions can be drawn.

1. Hardness tests indicated that samples containing MoS_2_ and WS_2_ exhibited an increase in hardness with the addition of more layers and higher compaction pressure, reaching values up to 100% higher than samples containing only Fe, sintered and compacted at 350 MPa. In contrast, samples with h-BN showed a decrease in hardness of up to 50% as the amount of added h-BN increased, compared to pure Fe samples, regardless of grain size and compaction pressure.
2. Based on the evaluation of samples stored for six years under the same conditions, it can be concluded that samples containing h-BN are significantly better suited for storage; even after the entire storage period, they showed only traces of corrosion. Samples containing WS_2_, or even more so, MoS_2_, displayed clear signs of corrosion after just a few days, which worsened over time.
3. Self-lubricating materials for sintered bearings made from iron powder containing h-BN are highly suitable for storage, even though they have slightly lower hardness and a marginally higher coefficient of friction compared to other materials. However, materials with MoS_2_ or WS_2_ are not ideal for storage unless they are given additional corrosion protection.
4. The results showed that increasing the amount of lubricant additives (for WS_2_ and MoS_2_) led to higher hardness, lower porosity, and a reduced coefficient of friction. The decrease in porosity may be because the added lubricants act similarly to Kenolube, reducing friction between powder particles during compaction.
5. In the case of boron nitride (h-BN), these relationships are less clear, as increasing the amount of lubricant additives can lead to a decrease in hardness and, in some cases, to an increase in the coefficient of friction.

A comparison of the surface condition of samples stored in a laboratory under normal atmospheric conditions for six years shows that those containing h-BN are by far the most suitable for storage, with no significant corrosion visible. The situation is different for samples containing MoS_2_ and WS_2_, where visible corrosion changes occur and their severity depends on the amount of the layered additive.

The research showed that materials for self-lubricating sintered bearings based on iron powder containing h-BN are suitable for storage, while those with MoS_2_ or WS_2_ need additional corrosion protection.

## Figures and Tables

**Table 1:** Basic parameters of SC 100.40 iron powder (http://hoganas.com) (accessed on 1 July 2025).

Approx. Particle Size Range [µm]	Apparent Density [g/cm^3^]	Flow [s/50 g]	H_2_-Loss [%]	C [%]	Green Density [g/cm^3^]
45–150	2.45	32	0.14	<0.01	7.1

**Table 2:** Density and porosity of the samples [30].

Sample No.	Type of Sinter	Additive Content (% Mass)	Pressing Pressure (MPa)			
			250 MPa		350 MPa	
			Density (g/cm^3^)	Porosity (%)	Density (g/cm^3^)	Porosity (%)
1.	Fe	0	5.82 ± 4%	26.1 ± 4%	6.35 ± 2%	19.3 ± 2%
2.	Fe + h-BN 0.5 μm	0.5	5.93 ± 3%	23.7 ± 3%	6.34 ± 4%	18.5 ± 4%
3.	Fe + h-BN 1.5 μm	0.5	5.92 ± 2%	23.9 ± 2%	6.34 ± 5%	
4.	Fe + MoS_2_ 1.5 μm	0.5	5.79 ± 4%	26.2 ± 4%	6.37 ± 6%	18.9 ± 6%
5.	Fe + WS_2_ 0.6 μm	0.5			6.39 ± 5%	18.8 ± 5%
6.	Fe + h-BN 0.5 μm	2.5	5.78 ± 1%	22.2 ± 1%	6.13 ± 2%	17.4 ± 2%
7.	Fe + h-BN 1.5 μm	2.5	5.77 ± 5%	22.2 ± 5%	6.15 ± 3%	17.1 ± 3%
8.	Fe + MoS_2_ 1.5 μm	2.5	5.74 ± 3%	26.1 ± 3%	6.36 ± 6%	18.1 ± 6%
9.	Fe + WS_2_ 0.6 μm	2.5	5.99 ± 6%	23.8 ± 6%	6.52 ± 5%	17.1 ± 5%
10.	Fe + h-BN 0.5 μm	5	5.59 ± 3%	20.4 ± 3%	5.84 ± 2%	16.8 ± 2%
11.	Fe + h-BN 1.5 μm	5	5.56 ± 3%	20.8 ± 3%	5.86 ± 1%	16.5 ± 1%
12.	Fe + MoS_2_ 1.5 μm	5	5.69 ± 5%	25.7 ± 5%	6.30 ± 4%	17.8 ± 4%
13.	Fe + WS_2_ 0.6 μm	5	6.00 ± 3%	23.5 ± 3%	6.51 ± 3%	17.1 ± 3%

**Table 3:** Hardness test results.

Sample No.	Type of Sinter	Additive Content (% Mass)	Hardness HB HB2./62.5/15	
			250 MPa	350 MPa
1.	Fe	0	32.4 ± 6%	41.3 ± 4%
2.	Fe + MoS_2_ 1.5 μm	0.5	46.5 ± 6%	51.7 ± 4%
3.	Fe + WS_2_ 0.6 μm	0.5	41.5 ± 5%	45.4 ± 5%
4.	Fe + h-BN 0.5 μm	0.5	36.4 ± 8%	43.1 ± 6%
5.	Fe + h-BN 1.5 μm	0.5	32.7 ± 7%	41.3 ± 8%
6.	Fe + MoS_2_ 1.5 μm	2.5	51.5 ± 5%	73.4 ± 4%
7.	Fe + WS_2_ 0.6 μm	2.5	48.8 ± 3%	72.0 ± 4%
8.	Fe + h-BN 0.5 μm	2.5	26.1 ± 9%	29.3 ± 8%
9.	Fe + h-BN 1.5 μm	2.5	30.6 ± 8%	31.0 ± 6%
10.	Fe + MoS_2_ 1.5 μm	5	73.3 ± 3%	94.6 ± 4%
11.	Fe + WS_2_ 0.6 μm	5	69.1 ± 5%	77.6 ± 6%
12.	Fe + h-BN 0.5 μm	5	13.2 ± 9%	15.9 ± 7%
13.	Fe + h-BN 1.5 μm	5	19.4 ± 7%	19.7 ± 8%

**Table 4:** Average values of the friction coefficient and wear track width for samples without lubrication additives.

Sample	Friction Coefficient Values		Mean Values	Abrasion Trace Width [µm]		Mean Values [µm]
	Test 01	Test 02		Test 01	Test 02	
Fe (350 MPa)	0.559	0.555	0.557 ± 0.5%	244	250	247 ± 1.7%
Fe (250 MPa)	0.550	0.549	0.550 ± 0.1%	259	247	253 ± 3.4%

**Table 5:** The average values of the friction coefficient and wear scar width for MoS_2_ samples.

Sample	Friction Coefficient Values		Mean Value	Abrasion Trace Width [µm]		Mean Value [µm]
	Test 01	Test 02		Test 01	Test 02	
M_O_S_2_ 1.5 µm 0.5% (350 MPa)	0.487	0.472	0.480 ± 2.2%	222	229	226 ± 2.2%
M_O_S_2_ 1.5 µm 0.5% (250 MPa)	0.472	0.463	0.468 ± 1.4%	237	236	237 ± 0.3%
M_O_S_2_ 1.5 µm 2.5% (350 MPa)	0.474	0.453	0.464 ± 3.2%	195	195	195 ± 0%
M_O_S_2_ 1.5 µm 2.5% (250 MPa)	0.431	0.428	0.430 ± 0.5%	200	201	201 ± 0.4%
M_O_S_2_ 1.5 µm 5% (350 MPa)	0.434	0.440	0.437 ± 1%	187	192	190 ± 1.9%
M_O_S_2_ 1.5 µm 5% (250 MPa)	0.436	0.445	0.441 ± 1.4%	193	198	196 ± 1.8%

**Table 6:** The average values of the friction coefficient and wear scar width for WS_2_ samples.

Sample	Friction Coefficient Values		Mean Values	Abrasion Trace Width [µm]		Mean Values [µm]
	Test 01	Test 02		Test 01	Test 02	
WS_2_ 0.6 µm 0.5% (350 MPa)	0.486	0.468	0.477 ± 2.7%	234	236	235 ± 0.6%
WS_2_ 0.6 µm 0.5% (250 MPa)	0.487	0.486	0.487 ± 0.2%	242	237	240 ± 1.5%
WS_2_ 0.6 µm 2.5% (350 MPa)	0.482	0.461	0.472 ± 3.1%	218	211	215 ± 2.3%
WS_2_ 0.6 µm 2.5% (250 MPa)	0.450	0.455	0.453 ± 0.8%	219	217	218 ± 0.6%
WS_2_ 0.6 µm 5% (350 MPa)	0.459	0.447	0.453 ± 1.9%	203	202	203 ± 0.3%
WS_2_ 0.6 µm 5% (250 MPa)	0.465	0.463	0.464 ± 0.3%	214	213	214 ± 0.3%

**Table 7:** The average values of the friction coefficient and the wear scar width for samples with h-BN 0.5 µm.

Sample	Friction Coefficient Values		Mean Values	Abrasion Trace Width [µm]		Mean Values [µm]
	Test 01	Test 02		Test 01	Test 02	
h-BN 0.5 µm 0.5% (350 MPa)	0.529	0.525	0.527 ± 0.5%	206	210	208 ± 1.4%
h-BN 0.5 µm 0.5% (250 MPa)	0.560	0.552	0.556 ± 1%	240	246	243 ± 1.7%
h-BN 0.5 µm 2.5% (350 MPa)	0.580	0.579	0.580 ± 0.1%	270	262	266 ± 2.1%
h-BN 0.5 µm 2.5% (250 MPa)	0.566	0.553	0.560 ± 1.6%	281	260	271 ± 5.5%
h-BN 0.5 µm 5% (350 MPa)	0.913	0.915	0.914 ± 0.2%	2414	2353	2384 ± 1.8%
h-BN 0.5 µm 5% (250 MPa)	0.656	0.793	0.725 ± 13%	2231	2171	2201 ± 1.9%

**Table 8:** The average values of friction coefficient and wear scar width for samples with h-BN 1.5 µm.

Sample	Friction Coefficient Values		Mean Values	Abrasion Trace Width [µm]		Mean Values [µm]
	Test 01	Test 02		Test 01	Test 02	
h-BN 1.5 µm 0.5% (350 MPa)	0.569	0.553	0.561 ± 2%	195	196	196 ± 0.4%
h-BN 1.5 µm 0.5% (250 MPa)	0.551	0.562	0.557 ± 1.4%	200	205	202 ± 1.7%
h-BN 1.5 µm 2.5% (350 MPa)	0.492	0.478	0.485 ± 2%	198	190	194 ± 3%
h-BN 1.5 µm 2.5% (250 MPa)	0.411	0.457	0.434 ± 7.5%	204	205	205 ± 0.3%
h-BN 1.5 µm 5% (350 MPa)	0.438	0.446	0.442 ± 1.3%	300	232	266 ± 18%
h-BN 1.5 µm 5% (250 MPa)	0.485	0.466	0.476 ± 2.8%	207	220	214 ± 4.3%

## Data Availability

The original contributions presented in this study are included in the article/Supplementary Material. Further inquiries can be directed to the corresponding author(s).

## Supplementary Materials

The following supporting information can be downloaded at: https://www.mdpi.com/article/10.3390/ma18174211/s1, Figure S1: h-BN—electron microscope photo; Figure S2: A crystallographic lattice diagram; Figure S3: MoS_2_—electron microscope photo; Figure S4: A crystallographic lattice diagram; Figure S5: WS_2_—electron microscope photo, Figure S6: A crystallographic lattice diagram; Figure S7: Fritsch ball mill; Figure S8: LPR 250 Hydraulic Press; Figure S9: Tube furnace RO 13.5; Figure S10. UHT 910 Tester; Figure S11. UNMT set; Figure S12: Drive for reciprocating motion tests; Figure S13: Nikon ECLIPSE LV100 microscope; Table S1: Important properties of h-BN; Table S2: Important properties of MoS_2_; Table S3: Important properties of WS_2_.
