# Supplementary material for: The Influence of Selected Properties of Sintered Iron Doped with Lubricants on Its Tribological Properties
Source: Materials (Basel). 2025 Sep 8;18(17):4211. doi: 10.3390/ma18174211 (PMC12430188; doi:10.3390/ma18174211)
Supplement: Supplementary file 1 [file materials-18-04211-s001.zip › materials-3819837-supplementary.pdf]

materials

Supplementary Material

for

Studies on the influence of selected properties and chemical composition of sintered iron with the addition of lubricants on the tribological properties of these materials

Wiesław Urbaniak, Tomasz Majewski, Grzegorz Śmigielski, Anna Trynda and Aneta D. Petelska

TABLE OF CONTENT

Materials and Methods

1. Testing materials S2
1.1. Lubricant additives S2
2. Measuring apparatus used for research S3

Supplementary tables and figures

Table S1. Important properties of h-BN. S2
Table S2. Important properties of MoS_2. ......S2
Table S3. Important properties of WS_2. ......S2

Figure S1. h-BN - electron microscope photo. S2
Figure S2. A **c**rystallographic lattice diagram. S2
Figure S3. MoS_2 - electron microscope photo. S2
Figure S4. A **c**rystallographic lattice diagram. S2
Figure S5. WS_2 - electron microscope photo. S2
Figure S6. A **c**rystallographic lattice diagram. S2
Figure S7. Fritsch ball mill. S3
Figure S8. LPR 250 Hydraulic Press. S3
Figure S9. Tube furnace RO 13.5. ......S4
Figure S10. UHT 910 Tester. S4
Figure. S11. UNMT set S5
Figure S12. Drive for reciprocating motion tests. S5
Figure S13. Nikon ECLIPSE LV100 microscope S5

Materials and Methods

1. Testing materials

1.1. Lubricant additives

Table S1. Important properties of h-BN.

Properties of hexagonal boron nitride	
Molecular weight (g/mol)	24.82
Metallic properties	non-metal
Appearance	white
Mohs hardness	1.5÷2
Density (g/cm^3)	1.7÷2.2
Melting point (°C)	2973
Lubrication temperature range (°C)	-40÷870
Friction coefficient	0.15÷0.7

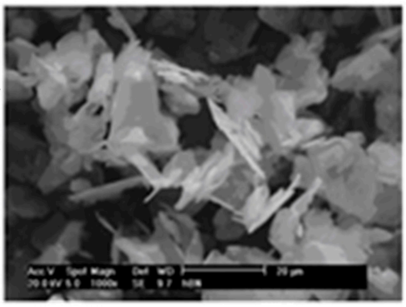

Figure S1. h-BN—electron microscope photo [1].

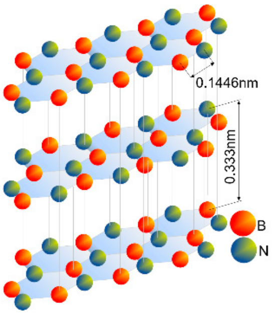

Figure S2. A crystallographic lattice diagram.

Table S2. Important properties of MoS_2.

Properties of molybdenum disulfide	
Molecular weight (g/mol)	160.9
Metallic properties	non-metal
Appearance	dark
Mohs hardness	1÷1.5
Density (g/cm^3)	5.06
Melting point (°C)	1185
Lubrication temperature range (°C)	185÷350
Friction coefficient	0.19

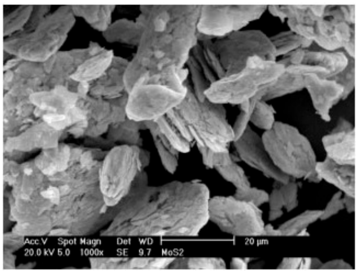

Figure S3. MoS_2—electron microscope photo [1].

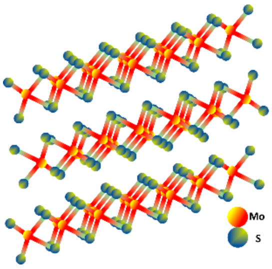

Figure S4. A crystallographic lattice diagram.

Table S3. Important properties of WS_2.

Properties of tungsten disulfide	
Molecular weight (g/mol)	248
Metallic properties	non-metal
Appearance	grey
Mohs hardness	0.5÷0.75
Density (g/cm^3)	7.5
Melting point (°C)	1250
Lubrication temperature range (°C)	od -273 to 650
Friction coefficient	0.07

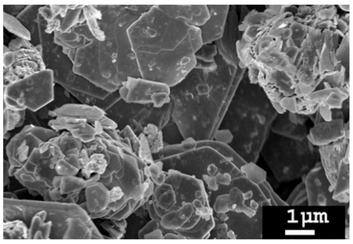

Figure S5. WS_2—electron microscope photo [1].

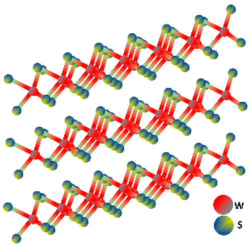

Figure S6. A crystallographic lattice diagram.

[1] Urbaniak, W. Smarowanie powierzchni biologicznych i inżynieryjnych występujących w strukturze warstwowej. UKW: Bydgoszcz, Poland, 2015. (In polish) [Google Scholar]

2. Measuring apparatus used for research

The following devices were used during the preparation of research samples and in the research process.

Ball mill

To prepare the powder for pressing, a Fritsch-Pulverisette ball mill, type 05-202, manufactured by Fritsch (Germany), was used (Figure S7).

Ball mill type 05-202	
Manufacturer	Fritsch-Pulverisette
Dimensions	5800x670x570 mm
Final fragmentation	<1 μm
Capacity swinging vessels	80, 250, 500 ml
Power supply	100-120/200-240 ~, 50-60 Hz
Rotational speed	50 - 400 rpm
Weight	100 kg

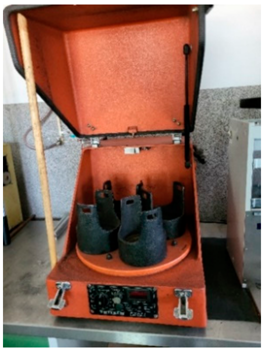

Figure S7. Fritsch ball mill.

Hydraulic press

The pressing of the test samples was carried out on an LPR 250 hydraulic press (Figure S8).

LPR 250 laboratory hydraulic press	
Manufacturer	Testchem Sp. z o.o.
Dimensions	270x400x300 mm
Maximum pressure	250kN
Workspace dimensions	Do 110 mm
Actuator stroke	120 mm
Diameter of pressed samples	10-40 mm

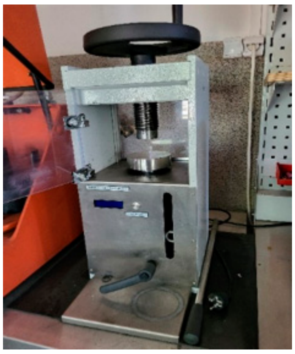

Figure S8. LPR 250 Hydraulic Press.

Tube furnace

The sintering process was carried out in a RO 13.5 tube furnace from VEB Elektro-Industrieofenbau Römhild (Figure S9), capable of reaching temperatures up to 1350 ^{0}C, with an operating power of 13.5 kW and the option to use a protective atmosphere during the sintering process.

**Tube furnace RO 13.5**	
Producent	VEB Elektro-Industrieofenbau Römhild
Operating temperature	1350°C
Electrical power	13.5kW
Pipe diameter	100 mm
Heating section length	740 mm
Temperature zone	single/double/triple

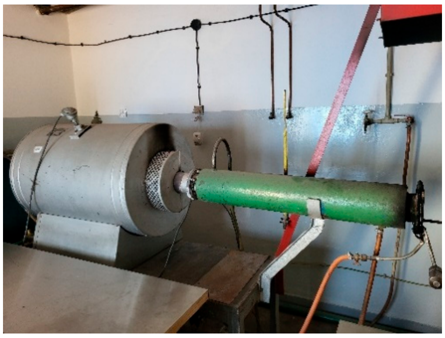

Figure S9. Tube furnace RO 13.5.

PRIDE UHT 910 Hardness Tester

The hardness measurement was carried out using a PRIDE UHT 910 hardness tester (Figure S10), which enables various hardness assessment procedures, including Rockwell Regular, Rockwell Superficial, Vickers, Vickers depth, Brinell, and Brinell depth.

**Universal UHT 910 Hardness Tester**	
Manufacturer	Eagle Eyes Quality Inspection Co., Ltd
Hardness scale	Brinell, Vickers, Rockwell
Standard compliance	ISO 6506, ISO 6507, ISO 6508, ASTM E18, ASTM E92, ASTM E10
Measuring height	to 300 mm
Clearance	to 150 mm
Simple shape	Cylindrical to 3 mm
Indenter types	Brinell Balls, Vickers Diamond, Rockwell Diamond Cone
Dimensions	250x567x1018 mm
Weight	200 kg

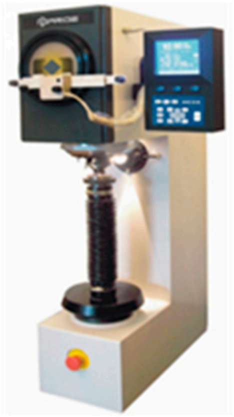

Figure S10. UHT 910 Tester.

UNMT Surface Layer Characterization Test Kit

Tribological studies were conducted using the UNMT Surface Layer Characterization Test Kit (Figure S11). The test drive (Figure S12) allows the system to perform tribological tests in the aforementioned rotary motion (0.1-1000 rpm) and reciprocating motion (0.1-25 mm stroke; 0.1-25 Hz frequency).

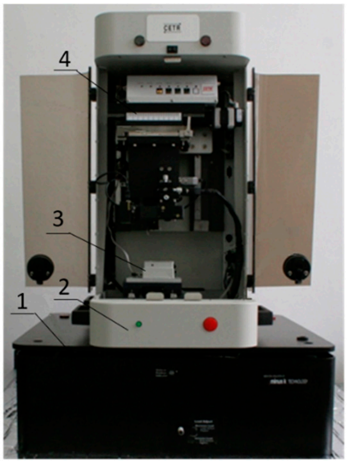

Figure S11. UNMT set, basic elements: 1—anti-vibration platform, 2— device body, 3— lower drive, 4—upper drive.

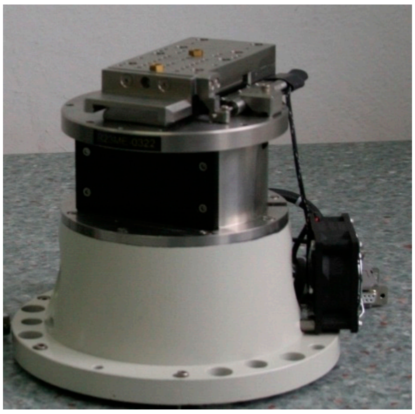

Figure S12. Drive for reciprocating motion tests.

Nikon ECLIPSE LV100 Optical Microscope

Microscopic experiments of the surface condition were performed using a Nikon ECLIPSE LV100 optical microscope (Figure S13) equipped with NIS-AR image analyzer computer software.

**Nikon ECLIPSE LV100 Microscope**	
Manufacturer	Nikon
Optical system	CF16 - chromatic aberration-free infinity system
Magnification	from 40 to 1500 x
Eyepiece	10x field number:22
Light source	50W halogen lighting (Fly-eye)
Focus range	to 30mm
Lenses	CFI P Achromat 4x,10,20x,40x,100x<br>CFI LU Plan Fluor Epi 5x,10x,20x,50x,100
Input ratings	Input voltage: 100-240 VAC- 50/60HZ<br>Rate current: 1.2A maximum

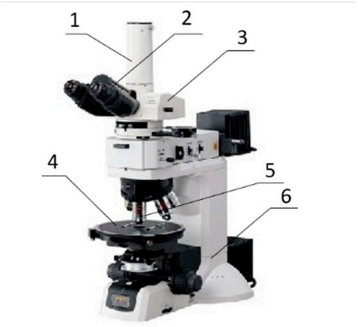

Figure S13. Nikon ECLIPSE LV100 microscope

1 – vertical tube adapter, 2- binocular section, 3- trinocular eyepiece tube, 4- measuring table, 5 – objective, 6- microscope body

Disclaimer/Publisher's Note: The statements, opinions and data contained in all publications are solely those of the individual author(s) and contributor(s) and not of MDPI and/or the editor(s). MDPI and/or the editor(s) disclaim responsibility for any injury to people or property resulting from any ideas, methods, instructions or products referred to in the content.
